# Endoloop-Assisted Polypectomy for a Symptomatic Giant Colonic Polyp in a Pediatric Patient

**DOI:** 10.3390/children9020222

**Published:** 2022-02-07

**Authors:** Yen-Chung Lin, Jen-Wei Chou, An-Chyi Chen, Shu-Fen Wu, Ching-Tien Peng, Walter Chen, Chien-Heng Lin

**Affiliations:** 1 Division of Pediatric Gastroenterology, China Medical University Children’s Hospital, Taichung 404327, Taiwan; 0036a@yahoo.com.tw (Y.-C.L.); d0344@mail.cmuh.org.tw (S.-F.W.); chenwalt@yahoo.com (W.C.); 2 Division of Gastroenterology and Hepatology, Department of Internal Medicine, China Medical University Hospital, Taichung 404327, Taiwan; codecol@yahoo.com.tw; 3 School of Medicine, China Medical University, Taichung 404328, Taiwan; 4 Division of Pediatric Hemato-Oncology, China Medical University Hospital, Taichung 404327, Taiwan; d0062@mail.cmuh.org.tw; 5 Division of Pediatric Pulmonology, China Medical University Hospital, Taichung 404327, Taiwan; lch227@ms39.hinet.net

**Keywords:** endoloop, colonoscopy, juvenile polyp, polypectomy

## Abstract

Colonic polyps are a common cause of persistent bloody stools in pediatric patients. Such polyps are easily diagnosed by a barium study of the lower gastrointestinal tract or by colonoscopy. Polypectomies utilizing electric ligators are generally performed on pediatric patients, and such patients can be easily operated on. However, giant colonic polyps have been reported in pediatric patients. In the past, a laparotomy or laparoscopy would have been performed in some pediatric patients diagnosed with a giant colonic polyp; however, the large size, location, or position of the polyp would sometimes be too large or the location or position of the polyp would make successful operation difficult. In general, larger stumps with large feeding arteries are associated with larger colonic polyps. Therefore, if such a polyp is removed via electric polypectomy alone, there may be a higher risk of post-polypectomy bleeding from its stump. We report a case of a 14-year-old male patient who presented with a 2-month history of bloody stools. A giant juvenile colonic polyp was detected by colonoscopy in the transverse colon. Finally, we successfully removed the giant polyp by using endoloop-assisted polypectomy.

## 1. Case Presentation

A 14-year-old boy without any prior systemic disease was admitted due to a 2-month history of bloody stools. He also complained of loose stools sometimes mixed with blood and mucus. The patient reported no abdominal pain, vomiting, diarrhea, fever, or fatigue during this period. The patient had no family history of polyposis or inflammatory bowel disease.

A physical examination conducted in our outpatient department revealed a soft, non-tender abdomen; no palpable mass; and normal bowel sounds. A digital rectal examination of the patient revealed stool mixed with bloody mucus. We then arranged a sigmoidoscopy, which revealed some small colonic ulcers with hemorrhagic spots in the distal colonic area. Due to the patient’s intolerance of the severe pain caused by this procedure, we could not approach the more proximal area of the colon. Due to a clinical suspicion of inflammatory bowel disease, the patient was admitted for colonoscopy under general anesthesia.

Upon admission, the patient’s general examination results were as follows. Activity: fair; consciousness: alert; mental status: alert and well-oriented; measurements—body weight: 62.4 kg (50–85th percentile); height: 178 cm (85–97th percentile); general appearance: acutely ill; vital signs—BT: 36.8 °C; HR: 88/min; RR: 20/min; BP: 114/76 mmHg; perfusion and oxygenation: warm, no cyanosis; head, eyes, ear, nose and throat exam (HEENT)—head: no facial dysmorphism; eyes—conjunctiva: pale; sclera: non-icteric; ears: no injection, no discharge; nose: negative; throat: not injected, no vesicles, no ulcers; neck: no lymphadenopathy; chest: no subcostal or suprasternal retraction; breathing sound: bilateral clear breathing sound; heart: normal sinus rhythm, no heart murmur; abdomen: soft, normoactive bowel sounds, no palpable mass or hepatosplenomegaly, no muscle guarding, no tenderness or rebound tenderness; extremities: freely movable; neurological examination: negative; anus: patent, rectal digital examination showed stool mixed with bloody mucus; genitalia: normal in appearance; back: grossly normal. Overall, all systems except for the large colon were unremarkable upon review.

## 2. Laboratory Results and Treatment

**Figure 1:**
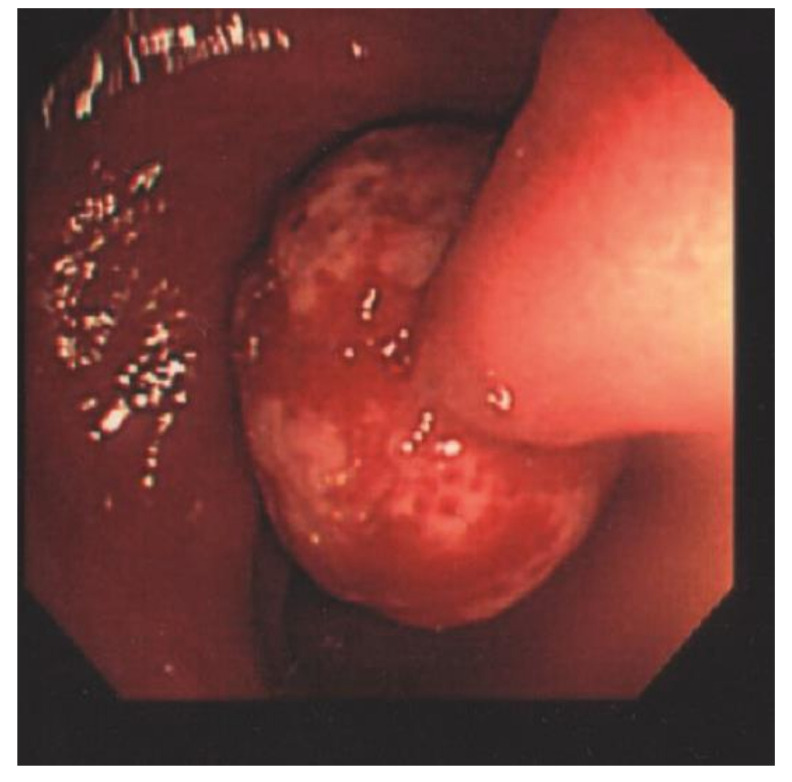
A huge Yamada type C colonic polyp, measuring 4 × 6 cm in diameter with an erosive surface and thick stalk, was detected in the transverse colon.

**Figure 2:**
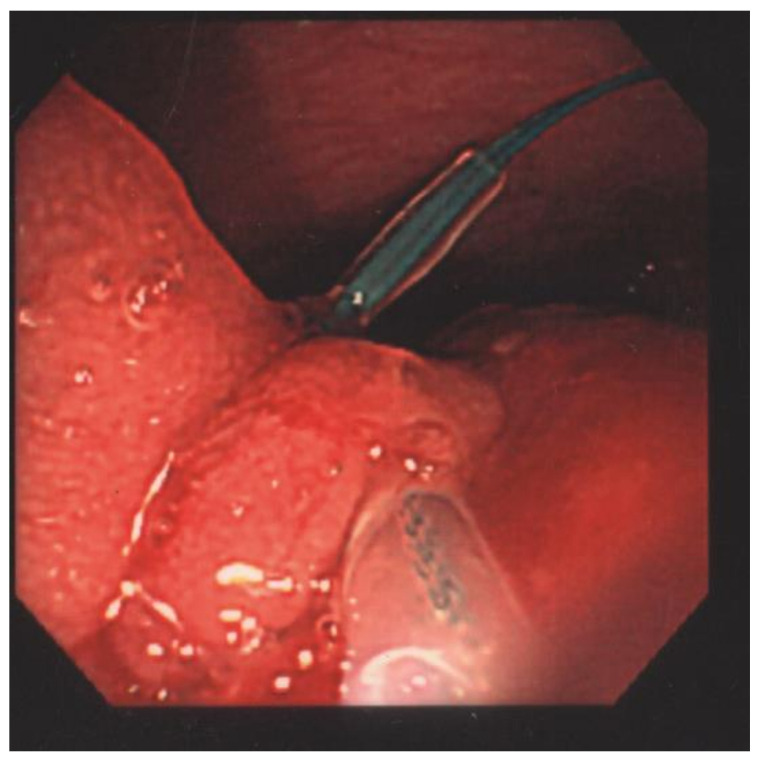
An endoloop (MAJ-254, Olympus, Tokyo, Japan) was used to ligate the stalk of the polyp to prevent post-polypectomy bleeding.

**Figure 3:**
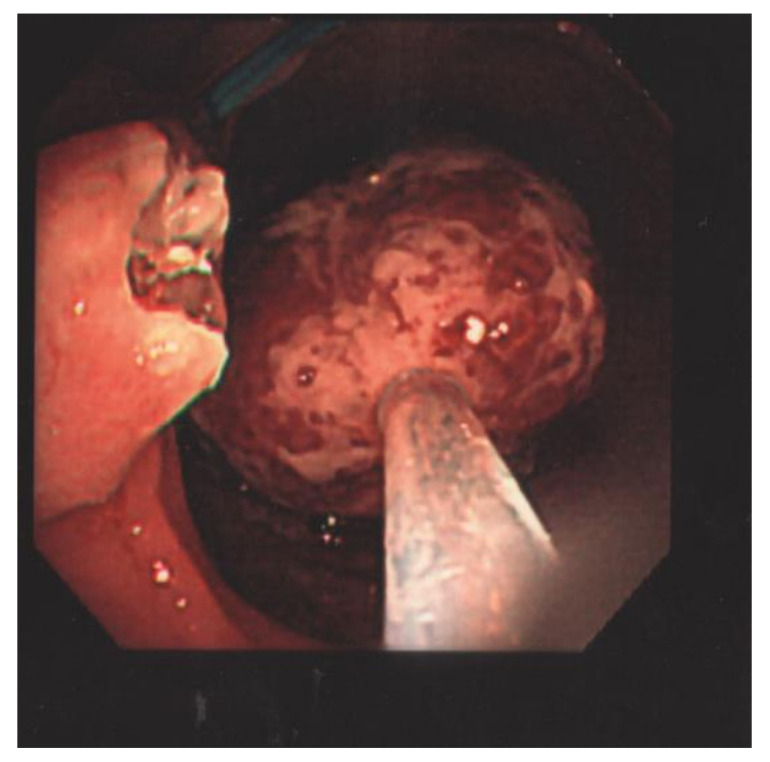
After successful looping of the tumor, we performed a polypectomy using an electrosurgical snare.

**Figure 4:**
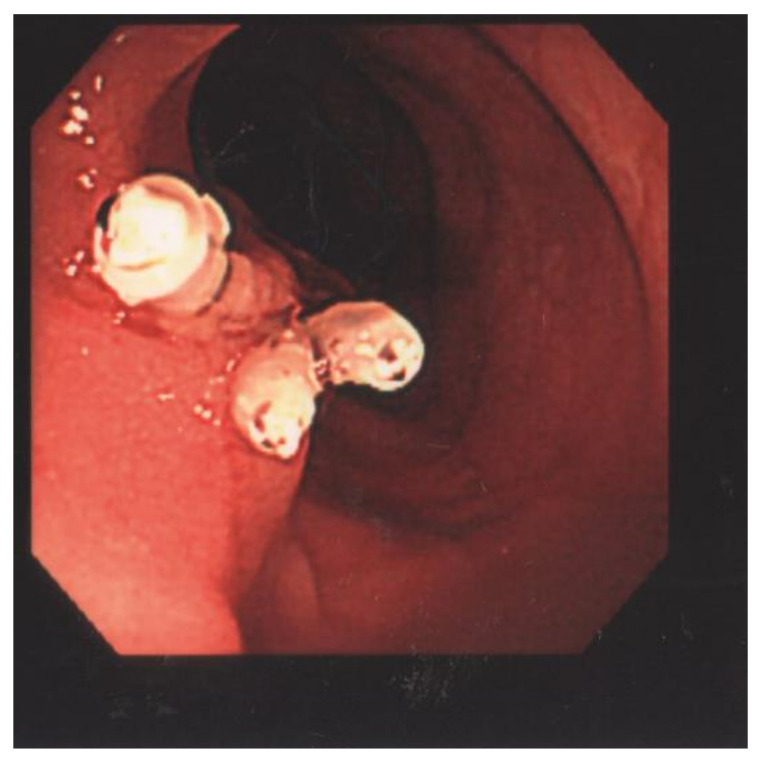
A large feeding vessel was identified on the cut surface of the tumor’s stalk and three hemoclips were utilized to clamp the vessel.

**Figure 5:**
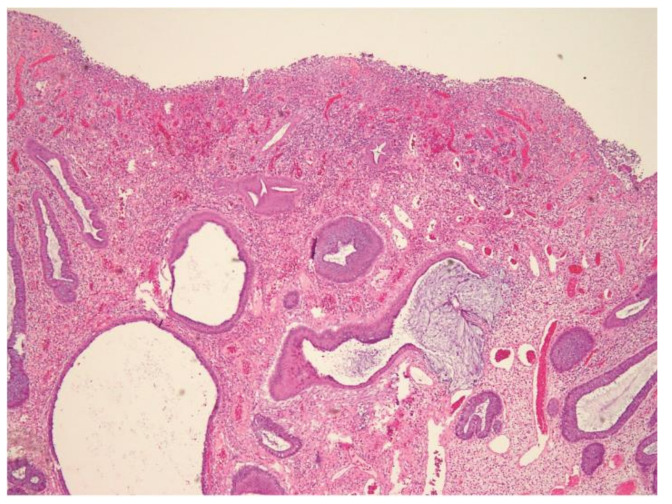
Pathohistological examination of the resected polyp showed evident surface erosions, significant lamina propria edema and stromal fibroplasia, scattered retention microcysts, and indistinct glandular dysplasia.

The laboratory data revealed a white blood cell count of 5120 cells/mm^3^, Hb of 14.4 g/dL, Hct of 40.6%, RBC of 5.50 × 106/uL, MCV of 73.8 fL, MCH of 26.2 pg, MCHC of 35.5 g/dL, RDW of 13.0%, LDH of 148 U/L, and hCRP of 0.06 mg/dL. The patient underwent a colonoscopy examination under general anesthesia after colonic preparation by oral fleet solution. Under general anesthesia, the patient was placed in the left decubitus position. The colonic scope was smoothly inserted into the anus until it reached the terminal ileum. A giant Yamada type C colonic polyp with an erosive surface, measuring 4 × 6 cm in diameter, was detected in the transverse colon (Figure 1). The polyp had a thick and long stalk. We considered the polyp to be the etiology of the bloody stools and decided to remove it to prevent further bleeding. Initially, we used an endoloop (MAJ-254, Olympus, Tokyo, Japan) to ligate the stalk of the polyp to prevent post-polypectomy bleeding (Figure 2). After successful looping of the tumor, we performed a polypectomy using an electrosurgical snare (Figure 3). There was no obvious postoperative bleeding from this procedure. However, a large feeding vessel was identified upon cutting the tumor’s stalk; therefore, we applied three hemoclips to clamp the vessel and prevent further bleeding (Figure 4). The pathohistological examination of the resected polyp showed evident surface erosions, significant lamina propria edema and stromal fibroplasia, scattered retention microcysts, and indistinct glandular dysplasia (Figure 5). No evidence of malignancy was seen in any of the sections. A diagnosis of sporadic juvenile polyp was made based on the pathohistological characteristics. Therefore, the patient was discharged with an uneventful course. Several days later, the patient was followed up at our outpatient department and appeared to be in good condition without further bleeding.

## 3. Discussion

Bloody stools are a common presentation in pediatric gastroenterology clinics. The most common cause of blood in the stool is anal fissure, followed by infectious enterocolitis and, in rare cases, colorectal polyps [1]. In the present case, we could easily exclude anal fissure and infectious enterocolitis as etiologies based on the patient’s history, physical examination, and laboratory data. The diagnosis of a colorectal polyp is conventionally made via a barium study of the lower gastrointestinal tract or a colonoscopy [2,3]. We performed a colonoscopy on this patient in an operating room following the administration of general anesthesia. A giant Yamada type C polyp, measuring 6 × 5 cm in diameter with a large stalk, was detected in the transverse colon during the colonoscopy. The colonic lumen was nearly impacted by the tumor. We considered using an electric ligator to cut the stump; however, to minimize the risk of stump bleeding, an endoloop-assisted polypectomy was performed, which was determined to be the best choice in this case [4,5,6] because an endoloop can tightly grip the stump and compress the associated feeding vessel [7,8,9]. No further bleeding was observed after the endoloop-assisted polypectomy of the big polyp. The presence of a large feeding artery in the center of the stump increased the risk of delayed bleeding upon loosening of the endoloop. There may have been some risk of delayed bleeding when the endoloop was loosened. Therefore, we applied three hemoclips to clamp the vessel smoothly [10]. We successfully managed the giant colonic polyp using this technique and completed the procedure without any complications.

Several endoscopic techniques have been developed to treat giant colorectal polyps, including the submucosal injection of saline or epinephrine, electrosurgical snare polypectomy, endoclipping, and endoloop-assisted polypectomy with or without snare resection. The endoloop, a detachable snare made of nylon that was originally developed by Tadashi Hachisu, has been used successfully to avoid post-polypectomy complications [5]. Previous reports regarding the treatment of giant colorectal polyps using endoloop-assisted polypectomy usually involved adult patients. Furthermore, physicians must acquire a high level of skill and considerable experience before attempting such procedures. Furthermore, the use of an endoloop-assisted polypectomy to treat a giant colorectal polyp in a pediatric patient has not been previously reported. Nonetheless, as demonstrated in the present case study, this technique can be applied in pediatric patients to reduce the risk of delayed bleeding after electrosurgical polypectomy and to ultimately decrease the need for open surgery.

In conclusion, this report describes a novel endoscopic technique used to safely and effectively treat a giant symptomatic juvenile colonic polyp. Endoloop-assisted polypectomy appears to be a promising new technique for the management of pediatric patients with giant colorectal polyps.
